# Phytohormone Profiling across the Bryophytes

**DOI:** 10.1371/journal.pone.0125411

**Published:** 2015-05-14

**Authors:** Lenka Záveská Drábková, Petre I. Dobrev, Václav Motyka

**Affiliations:** 1 Department of Taxonomy, Institute of Botany, Academy of Sciences of the Czech Republic, Průhonice, Czech Republic; 2 Laboratory of Hormonal Regulations in Plants, Institute of Experimental Botany, Academy of Sciences of the Czech Republic, Prague, Czech Republic; University of Nottingham, UNITED KINGDOM

## Abstract

**Background:**

Bryophytes represent a very diverse group of non-vascular plants such as mosses, liverworts and hornworts and the oldest extant lineage of land plants. Determination of endogenous phytohormone profiles in bryophytes can provide substantial information about early land plant evolution. In this study, we screened thirty bryophyte species including six liverworts and twenty-four mosses for their phytohormone profiles in order to relate the hormonome with phylogeny in the plant kingdom.

**Methodology:**

Samples belonging to nine orders (Pelliales, Jungermanniales, Porellales, Sphagnales, Tetraphidales, Polytrichales, Dicranales, Bryales, Hypnales) were collected in Central and Northern Bohemia. The phytohormone content was analysed with a high performance liquid chromatography electrospray tandem-mass spectrometry (HPLC-ESI-MS/MS).

**Principal Findings:**

As revealed for growth hormones, some common traits such as weak conjugation of both cytokinins and auxins, intensive production of *cis*Z-type cytokinins and strong oxidative degradation of auxins with abundance of a major primary catabolite 2-oxindole-3-acetic acid were pronounced in all bryophytes. Whereas apparent dissimilarities in growth hormones profiles between liverworts and mosses were evident, no obvious trends in stress hormone levels (abscisic acid, jasmonic acid, salicylic acid) were found with respect to the phylogeny.

**Conclusion:**

The apparent differences in conjugation and/or degradation strategies of growth hormones between liverworts and mosses might potentially show a hidden link between vascular plants and liverworts. On the other hand, the complement of stress hormones in bryophytes probably correlate rather with prevailing environmental conditions and plant survival strategy than with plant evolution.

## Introduction

Bryophytes are a very diverse group of non-vascular land plants with over 800 genera and 12 thousand species which include liverworts, mosses and hornworts [1]. After the colonization of land by an ancestor most closely related to modern day charophycean algae [2], bryophytes arose during the Ordovician, ca 470 million years ago. The fossil record of bryophytes is rather sketchy. Therefore their phylogeny is based on molecular sequence data and morphology of the extant species. The liverworts (Marchantiophyta) are resolved as the earliest-divergent land plant group [3], while the mosses (Bryophyta) represent the sister group to a clade formed by hornworts (Anthroceratophyta) and vascular plants (Tracheophyta). The controversial hypothesis, less well supported, resolved hornworts as sister to mosses plus vascular plants [4]. Nevertheless, hypotheses are changing as new data accumulate.

**Fig 1:**
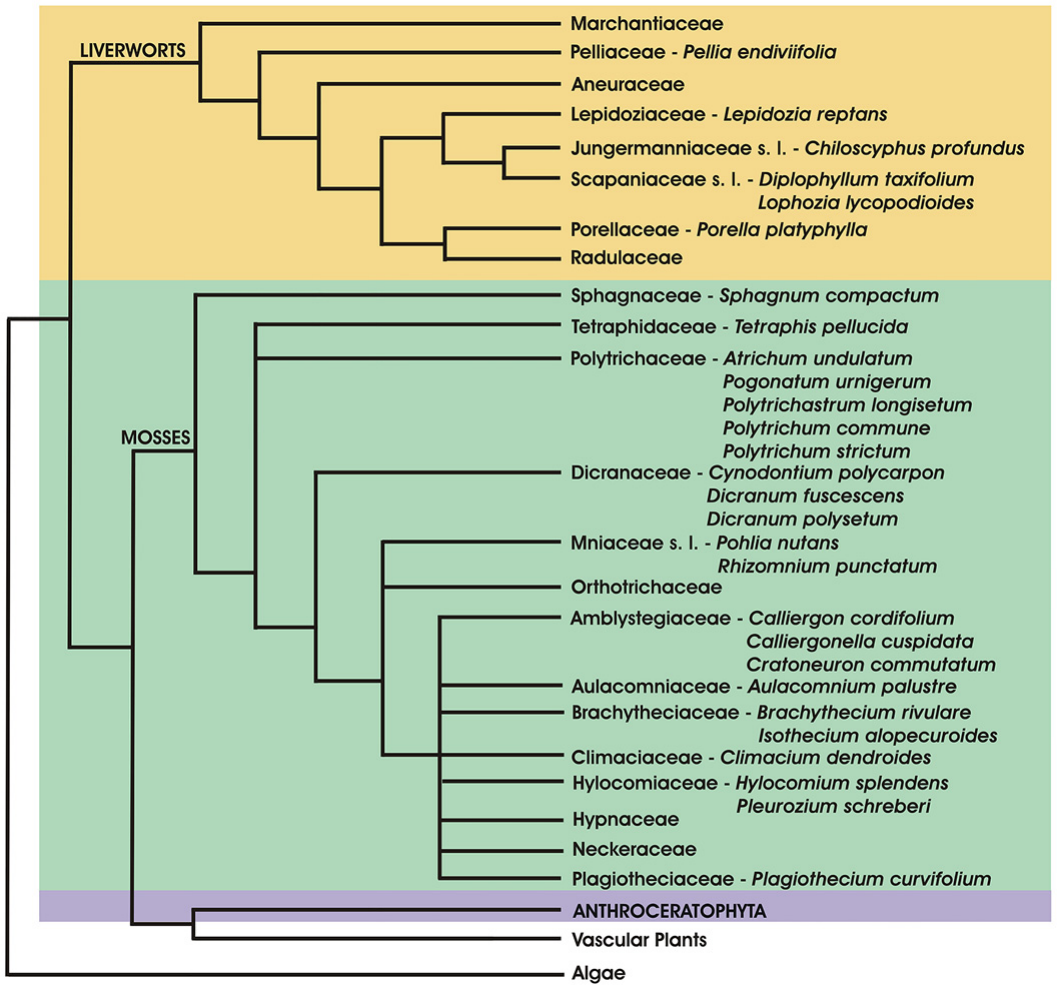
Simplified phylogenetic tree of bryophytes with selected representatives used in the study. For complete list of analyzed species see S1 Table.

The transition of plants from water to land was accompanied by major innovations. As the only land plants with a dominant gametophyte generation, bryophytes exhibit structural and reproductive attributes that are exclusive, unifying, and innovative [5]. The bryophytes developed during a time in which gametophyte characteristics were important for plant survival, whereas the vascular plants evolved when conditions favoured the sporophyte portion of the life cycle. However, there is strong evidence that bryophytes are an artificial, non-monophyletic group (Fig 1). Instead, the liverworts, mosses, and hornworts appear to form an evolutionary grade leading to the vascular plants. Recent knowledge shows that the liverworts are the earliest lineage sister to all other groups of land plants, followed by the mosses, and the hornworts are sister to the vascular plants [6,7].

With their simple morphology including only few differentiation steps, on one hand, and their responsiveness to various plant growth regulators, on the other hand, bryophytes represent interesting model organisms for studying the evolution of plant hormones [8–11]. Plant hormones (phytohormones) are defined as naturally occurring organic substances that influence physiological processes at low (10^–6^ to 10^–9^ M) concentrations [12]. Five groups of compounds—auxins, cytokinins (CKs), gibberellins (GAs), abscisic acid (ABA) and ethylene—are usually referred to as the classic phytohormones while more recently discovered brassinosteroids (BRs), salicylic acid (SA), jasmonic acid (JA) and strigolactones have been added to the list.

In seed plants, phytohormones regulate crucial growth and developmental events such as germination, vegetative growth, flowering, seed development, senescence, dormancy, mobilization of nutrients and stress tolerance. Plant hormones are not restricted to seed plants alone but they have been found also in lower order plants, algae and bacteria (e.g. [13–17]). In bryophytes, only auxins and CKs have been so far investigated extensively. It does not, however, mean that other classes of plant hormones are absent or less important. There are numerous reports demonstrating their role coordinating growth and stress responses and regulating most physiological processes of the liverworts, mosses, and hornworts ([10,11,15] and references therein). The only exception seems to refer to GAs; it is still not known whether or not bryophytes produce them and experimental support for morphogenetic action in bryophytes is missing [10,11,15].

Most of the research in plant hormones in bryophytes is limited to the model moss *Physcomitrella patens* and, to a lesser extent, *Funaria hygrometrica*. The recent genomic sequence for *Physcomitrella patens* [18] has contributed to a deeper understanding of the organization and evolution of genes associated with phytohormone homeostasis and signal transduction pathways. However, there is only very scarce information regarding the occurrence, metabolism and function of plant hormones in other bryophyte species.

The aim of this study is to identify relationships between the hormonome and the phylogenetic position of bryophytes within the plant kingdom. Using high performance liquid chromatography electrospray tandem-mass spectrometry (HPLC-ESI-MS/MS) we analysed the phytohormone content of a number of bryophyte species including liverworts and mosses. Based on the obtained results it is suggested that the metabolic profiles of phytohormones in bryophytes might reveal a link between vascular plants and liverworts.

## Materials and Methods

### Plant material

Bryophyte samples were collected in forested areas of Central Bohemia, Křivoklátsko (three main localities: Řevničov, Prameny Klíčavy and U Eremita) and Northern Bohemia, Krkonoše Mts. (Kozí hřbety). In total, thirty samples were assembled, six liverworts (Marchantiophyta) from six families and twenty-four mosses (Bryophyta) from twelve families. As Central and Northern Bohemia localities differ in their natural conditions, the samples from higher altitudes in Krkonoše Mts. were collected later than those from lowland and upland to obtain tissues in comparable growth stages. The vegetative young leaves and new shoots on a branch were collected directly in natural conditions, put immediately into dry ice and stored at -80°C prior to phytohormone analyses. A list of the species analysed in this study together with geographic coordinates of particular sampling areas and dates of collection can be found in S1 Table. Permission to conduct the study on these sites was according to agreements between CAS and appropriate authorities in visited localities. Special permission OSS KRNAP 04781/2012 was obtained from Natural park of Krkonoše.

### Phytohormone analysis

The analysis of plant hormones was carried out as described in [19, 20]. An aliquot of about 100 mg fresh weight of frozen plant material was homogenized in liquid nitrogen by mortar and pestle. Cold extraction buffer (methanol/water/formic acid, 15/10/5, v/v/v, -20°C, 500 μL) was added to the plant homogenates together with a mixture of stable isotope labelled internal standards (10 pmol). The following internal standards were added: ^13^C_6_-indole-3-acetic acid (IAA; Cambridge Isotope Laboratories), ^2^H_4_-SA (Sigma-Aldrich), ^2^H_6_-ABA (NRC-PBI), ^2^H_3_-phaseic acid (PA; NRC-PBI), ^2^H_5_-JA (C-D-N Isotopes Inc.), ^2^H_5_-*trans*Z, ^2^H_5_-*trans*ZR, ^2^H_5_-*trans*Z7G, ^2^H_5_-*trans*Z9G, ^2^H_5_-*trans*ZOG, ^2^H_5_-*trans*ZROG, ^2^H_5_-*trans*ZRMP, ^2^H_3_-DHZ, ^2^H_3_-DHZR, ^2^H_3_-DHZ9G, ^2^H_6_-iP, ^2^H_6_-iPR, ^2^H_6_-iP7G, ^2^H_6_-iP9G, ^2^H_6_-iPRMP (all CK standards Olchemim; the system of CK abbreviations adopted and modified according to ([21], see S2 Table), ^2^H_3_-castasterone (Olchemim), ^2^H_3_-epibrassinolide (Olchemim), ^2^H_2_-GA_4_ (GA_4_), ^2^H_2_-GA_8_, ^2^H_2_-GA_19_ and ^2^H_2_-GA_20_ (all GA standards Olchemim). After incubation for 30 min at -20°C, the extract was centrifuged at 17 000 rpm and supernatant was collected. A second extraction of the residue followed and the pooled supernatants evaporated under vacuum (Alpha RVC, Christ). The sample was dissolved into 0.1 M formic acid and applied to mixed mode reversed-phase cation exchange SPE column (Oasis-MCX, Waters). Two fractions were eluted: fraction A with methanol—contained acidic and neutral compounds (auxins, GAs, BRs, ABA, SA, JA), and fraction B with 0.35 M NH_4_OH in 70% methanol—contained basic compounds (CKs). Fractions were evaporated to dryness in vacuum concentrator and dissolved in 10% methanol (30 μL). An aliquot (10 μL) from each fraction was separately analyzed on HPLC (Ultimate 3000, Dionex) coupled to hybrid triple quadrupole/linear ion trap mass spectrometer (3200 Q TRAP, Applied Biosystems) set in the selected reaction monitoring mode. Chromatographic conditions for fraction A: HPLC column Luna C18(2) (100 x 2 mm, 3 μm, Phenomenex), flow rate 0.25 mL/min, linear gradient of solvent A (5 mM ammonium formate, pH 3 in water) and solvent B (5 mM ammonium formate, pH 3, in acetonitrile) from 10% B to 50% B for 15 min. Chromatographic conditions for fraction B: HPLC column Luna C18(2) (150 x 2mm, 3 μm, Phenomenex), flow rate 0.25 mL/min, linear gradient of solvent A (5 mM ammonium acetate, pH 4 in water) and solvent B (5 mM ammonium acetate, pH 4, in methanol) from 10% B to 40% B for 20 min. Mass spectrometry was run at electrospray ionization mode, negative for fraction A, and positive for fraction B. Ion source parameters included: ion source voltage -4000 V (negative mode) or +4500 V (positive mode), nebulizer gas 50 psi, heater gas 60 psi, curtain gas 20 psi, heater gas temperature 500°C. Quantification of phytohormones was done using isotope dilution method with multilevel calibration curves (r^2^ > 0.99). Data processing was carried out with Analyst 1.5 software (Applied Biosystems).

### Presentation of phytohormone profiles

Each evaluation was carried out in duplicates in two independent experiments. The results of analyses of the two experiments were not possible to average due to the accidental decrease in the mass spectrometry momentary response during the sample analysis of the second experiment. Thus the results only of the first experiment are presented. They are expressed as mean values including standard deviation (SD) of the means and coefficient of variance (CV) (full details in S3, S4 and S5 Tables).

The data trends of both experiments were comparable, with almost identical phytohormone profiles detected as well as with the same or very similar interrelationships among individual derivatives. The SD and CV of the presented results (S3, S4 and S5 Tables) are within acceptable confidence values.

## Results

The samples of thirty bryophyte species including six liverworts and twenty-four mosses were collected in Central and Northern Bohemia (S1 Table) and prepared for endogenous phytohormone analyses. The selected bryophytes belonging to nine orders (Pelliales, Jungermanniales, Porellales, Sphagnales, Tetraphidales, Polytrichales, Dicranales, Bryales, Hypnales) were analyzed and their complete list and position within a simplified phylogenetic tree are shown in Fig 1 and S1 Table.

The hormone analysis was performed by the dual-mode solid-phase method and HPLC-electrospray tandem-mass spectrometry, which allowed simultaneous and highly reliable identification and quantification of over 40 phytohormone metabolites including growth hormones (CKs, auxins, GAs, BRs), stress hormones (ABA, JA, SA) and conjugates.

### Endogenous cytokinins

In total, 26 isoprenoid CKs were detected in various bryophyte samples including derivatives of *trans*Z, *cis*Z (7 forms each), iP and DHZ (6 forms each). Total CK concentrations varied in different species ranging from picomols (e.g. *Sphagnum compactum*, 5.75 pmol/g FW) to hundreds of picomols (e.g. *Polytrichastrum longisetum*, 201.11 pmol/g FW) (Table 1 and S3 Table).

**Table 1:** Distribution and endogenous levels (pmol/g FW) of different cytokinin (CK) types in bryophytes presented on the basis of their *N* ^6^-side chain structure.

Division	Order	Family	Species	*trans*Z	*trans*ZR	*trans*Z7G	*trans*Z9G	*trans*ZOG	*trans*ZROG	*trans*ZRMP	∑ *trans*Z types	DHZ	DHZR	DHZ7G	DHZ9G	DHZROG	DHZRMP	∑ DHZ types	*cis*Z	*cis*ZR	*cis*Z7G	*cis*Z9G	*cis*ZOG	*cis*ZROG	*cis*ZRMP	∑ *cis*Z types	iP	iPR	iP7G	iP9G	iPRMP	∑ iP types	∑ total CKs
MARCHANTIOPHYTA (Liverworts)	Pelliales	Pelliaceae	*Pellia endiviifolia*	0,41	0,16	0,07	0,02	0,00	0,01	0,16	**0,84**	0,22	0,00	n. d.	0,01	0,02	0,13	**0,38**	0,96	0,90	0,01	0,18	0,02	0,18	2,28	**4,53**	0,51	1,62	1,09	1,70	0,69	**5,61**	**11,35**
	Jungermaniales	Lepidoziaceae	*Lepidozia reptans*	0,36	0,16	0,19	0,01	0,02	0,12	0,18	**1,04**	3,28	0,06	n. d.	0,25	0,11	0,38	**4,08**	0,77	0,94	n. d.	n. d.	0,03	0,49	1,45	**3,67**	1,57	0,70	0,45	0,25	0,84	**3,80**	**12,59**
	Jungermanniales	Lophocoleaceae	*Chiloscyphus profundus*	1,37	0,15	0,04	0,20	0,15	0,45	0,14	**2,51**	0,36	0,02	n. d.	0,02	0,01	0,33	**0,73**	1,77	0,32	0,04	0,03	0,15	4,65	3,61	**10,57**	2,39	0,39	2,22	0,06	1,27	**6,34**	**20,15**
	Jungermanniales	Scapaniaceae	*Diplophyllum taxifolium*	1,04	0,21	0,06	0,10	0,25	0,20	2,34	**4,21**	0,28	0,22	0,01	0,01	0,18	0,39	**1,08**	0,67	1,85	0,04	0,02	0,04	0,73	0,59	**3,94**	0,46	0,42	5,11	0,11	0,65	**6,74**	**15,97**
	Jungermanniales	Lophoziaceae	*Lophozia lycopodioides*	1,08	0,14	0,02	0,25	0,36	0,12	0,61	**2,58**	0,73	0,31	0,03	0,02	0,13	0,07	**1,30**	0,28	2,98	0,07	0,07	0,02	1,75	0,06	**5,22**	0,99	0,48	1,52	0,22	0,95	**4,16**	**13,26**
	Porellales	Porellaceae	*Porella platyphylla*	0,53	0,08	0,14	0,27	0,10	0,69	0,36	**2,18**	0,25	0,01	n. d.	0,16	1,36	0,01	**1,79**	1,82	0,59	0,03	0,04	0,02	1,27	6,56	**10,33**	1,47	1,05	0,53	0,21	1,19	**4,45**	**18,76**
BRYOPHYTA (Mosses)	Sphagnales	Sphagnaceae	*Sphagnum compactum*	0,50	0,05	0,01	0,01	0,05	0,00	0,10	**0,72**	0,06	0,09	0,01	0,01	0,01	0,20	**0,39**	0,10	0,13	0,01	0,00	0,01	0,15	0,20	**0,61**	0,17	0,76	1,24	0,25	1,62	**4,04**	**5,75**
	Sphagnales	Sphagnaceae	*Sphagnum sp*.	0,41	0,19	0,05	0,08	0,09	0,01	0,12	**0,95**	0,04	0,01	n. d.	0,05	0,03	0,01	**0,15**	0,11	0,27	0,03	0,01	0,01	0,01	0,14	**0,59**	0,37	0,43	3,24	0,06	4,93	**9,03**	**10,72**
	Tetraphidales	Tetraphidaceae	*Tetraphis pellucida*	0,80	0,18	0,07	0,18	1,70	8,62	0,16	**11,72**	0,81	0,05	n. d.	0,06	1,16	0,13	**2,21**	2,46	1,63	n. d.	n. d.	0,31	17,67	1,15	**23,22**	9,20	3,40	6,45	0,13	3,21	**22,39**	**59,53**
	Polytrichales	Polytrichaceae	*Atrichum undulatum*	0,93	0,40	0,11	0,15	0,31	1,85	0,38	**4,12**	0,23	0,06	n. d.	0,17	0,45	0,72	**1,64**	3,10	3,81	n. d.	n. d.	1,02	33,00	7,71	**48,63**	2,21	1,23	1,07	0,02	5,03	**9,55**	**63,95**
	Polytrichales	Polytrichaceae	*Pogonatum urnigerum*	2,85	0,51	0,04	0,16	0,82	1,59	2,13	**8,10**	1,37	0,47	0,01	0,13	0,33	6,90	**9,20**	5,90	4,38	0,03	0,44	0,07	19,03	4,51	**34,35**	1,95	4,87	0,71	0,14	2,55	**10,21**	**61,87**
	Polytrichales	Polytrichaceae	*Polytrichastrum longisetum*	2,21	0,19	0,03	0,18	0,98	11,36	3,61	**18,57**	0,88	0,25	0,07	0,07	4,57	63,87	**69,71**	8,30	3,97	0,07	0,06	0,97	79,69	4,68	**97,75**	6,47	2,87	2,57	0,12	3,05	**15,08**	**201,11**
	Polytrichales	Polytrichaceae	*Polytrichum commune*	0,87	0,25	0,06	0,22	0,89	5,18	0,23	**7,70**	0,15	0,06	n. d.	0,08	1,21	0,08	**1,57**	1,87	4,40	0,07	0,06	0,03	29,56	7,58	**43,57**	4,97	2,95	0,56	0,26	6,64	**15,38**	**68,22**
	Polytrichales	Polytrichaceae	*Polytrichum strictum*	2,08	0,18	0,03	0,09	0,55	0,76	2,98	**6,67**	1,01	0,21	0,07	0,21	0,69	3,10	**5,28**	9,19	2,99	0,02	0,02	0,07	4,36	3,21	**19,87**	5,78	1,80	1,98	0,18	2,74	**12,48**	**44,31**
	Dicranales	Dicranaceae	*Cynodontium polycarpon*	0,99	0,25	0,06	0,14	0,83	0,23	0,18	**2,69**	0,38	0,06	0,28	0,19	0,82	0,16	**1,90**	11,63	0,49	0,07	0,05	0,33	28,49	0,08	**41,14**	3,67	0,49	4,37	0,23	1,43	**10,19**	**55,92**
	Dicranales	Dicranaceae	*Dicranum fuscescens*	1,43	0,09	0,01	0,07	0,10	0,34	0,15	**2,18**	0,49	0,09	0,15	0,03	0,17	0,10	**1,04**	3,61	0,41	0,01	0,01	0,01	8,77	0,17	**13,00**	6,06	1,81	3,92	0,15	1,79	**13,72**	**29,95**
	Dicranales	Dicranaceae	*Dicranum polysetum*	0,99	0,15	0,05	1,83	0,29	0,31	0,03	**3,66**	0,38	0,02	n. d.	0,44	0,03	0,08	**0,94**	2,43	0,20	n. d.	n. d.	0,35	3,37	0,37	**6,72**	2,64	1,14	0,25	0,42	0,59	**5,03**	**16,34**
	Dicranales	Dicranaceae	*Dicranum sp*.	2,08	0,14	0,02	0,05	0,26	0,56	0,88	**3,99**	0,33	0,14	0,04	0,07	0,18	2,37	**3,13**	2,70	0,41	0,02	0,01	0,39	5,49	1,13	**10,16**	1,52	0,98	0,56	0,57	1,21	**4,84**	**22,11**
	Bryales	Mniaceae	*Pohlia nutans*	1,03	0,16	0,05	0,05	0,22	0,24	0,17	**1,92**	0,46	0,13	n. d.	0,01	0,58	0,08	**1,27**	32,53	1,78	0,02	0,02	0,03	11,35	0,18	**45,91**	5,99	3,61	2,27	5,71	3,36	**20,94**	**70,04**
	Bryales	Mniaceae	*Rhizomnium punctatum*	0,82	0,19	0,24	0,20	1,04	1,41	0,06	**3,96**	0,76	0,03	n. d.	2,03	1,17	0,05	**4,03**	6,52	4,06	n. d.	n. d.	3,07	31,25	1,93	**46,83**	1,10	0,97	0,88	0,10	2,93	**5,98**	**60,80**
	Hypnales	Amblystegiaceae	*Calliergon cordifolium*	1,95	0,40	0,14	0,77	0,29	3,48	0,57	**7,60**	0,93	0,89	0,02	0,21	3,20	0,13	**5,38**	5,74	1,56	0,02	0,02	2,48	67,08	2,84	**79,74**	3,09	3,27	1,96	3,54	16,56	**28,42**	**121,14**
	Hypnales	Amblystegiaceae	*Calliergonella cuspidata*	0,86	0,16	0,02	0,07	0,15	1,24	0,09	**2,59**	0,08	0,03	n. d.	0,03	0,60	0,13	**0,88**	4,41	0,33	n. d.	n. d.	2,02	41,20	1,09	**49,05**	6,74	2,02	0,25	0,09	2,93	**12,02**	**64,54**
	Hypnales	Amblystegiaceae	*Cratoneuron commutatum*	0,26	0,09	0,04	0,05	0,05	0,49	0,13	**1,10**	0,19	0,04	n. d.	0,01	0,23	0,13	**0,60**	0,53	0,73	0,00	0,01	0,01	4,94	0,29	**6,52**	0,36	1,17	0,52	0,02	0,82	**2,90**	**11,11**
	Bryales	Aulacomniaceae	*Aulacomnium palustre*	0,84	0,17	0,04	0,12	0,62	3,73	0,09	**5,60**	0,78	0,02	n. d.	0,13	3,59	0,13	**4,65**	10,66	1,16	n. d.	n. d.	4,19	83,81	0,80	**100,62**	1,96	3,45	2,57	0,63	23,49	**32,10**	**142,96**
	Hypnales	Brachytheciaceae	*Brachythecium rivulare*	1,01	0,41	0,08	0,11	3,37	1,93	1,34	**8,24**	0,30	0,03	n. d.	0,06	16,60	0,10	**17,08**	10,91	0,77	n. d.	n. d.	1,25	28,41	0,06	**41,39**	3,33	1,27	0,73	0,04	2,47	**7,85**	**74,57**
	Hypnales	Brachytheciaceae	*Isothecium alopecuroides*	0,81	0,11	0,05	0,01	0,01	1,21	0,08	**2,27**	0,22	0,02	n. d.	0,08	0,87	0,05	**1,25**	4,10	0,18	n. d.	n. d.	0,54	23,70	0,05	**28,57**	2,13	0,26	0,40	0,15	0,72	**3,66**	**35,74**
	Hypnales	Climaciaceae	*Climacium dendroides*	1,26	0,30	0,05	0,09	0,23	0,82	0,43	**3,18**	0,36	0,00	n. d.	0,04	0,32	0,18	**0,90**	9,30	0,72	n. d.	n. d.	1,00	13,44	0,69	**25,14**	5,28	1,91	1,15	0,06	2,84	**11,24**	**40,46**
	Hypnales	Hylocomiceae	*Hylocomium splendens*	0,47	0,12	0,04	0,12	0,14	3,62	0,22	**4,74**	0,21	0,03	n. d.	0,01	1,27	0,06	**1,59**	2,42	0,22	n. d.	n. d.	0,49	33,33	0,90	**37,35**	4,12	1,10	0,58	0,07	1,79	**7,65**	**51,33**
	Hypnales	Hylocomiaceae	*Pleurozium schreberi*	0,57	0,04	0,01	0,03	0,05	1,35	0,25	**2,30**	0,54	0,06	n. d.	0,03	0,78	0,14	**1,54**	4,02	0,27	n. d.	n. d.	0,41	27,08	2,34	**34,13**	4,16	1,12	0,09	0,05	2,25	**7,67**	**45,63**
	Hypnales	Plagiotheciaceae	*Plagiothecium curvifolium*	0,57	0,17	0,08	0,41	0,30	1,62	0,46	**3,63**	0,98	0,05	n. d.	0,11	0,46	0,18	**1,76**	5,23	0,64	n. d.	n. d.	1,32	32,62	1,57	**41,38**	5,53	1,24	1,19	0,42	3,53	**11,92**	**58,69**

CKs are divided into four groups including *trans*-zeatin (*trans*Z), dihydrozeatin (DHZ), *cis*-zeatin (*cis*Z) and *N* ^6^-(Δ^2^-isopentenyl)adenine (iP) types. The results are shown as mean values; for complete data containing standard deviations of the means and coefficients of variance see S3 Table.

**Fig 2:**
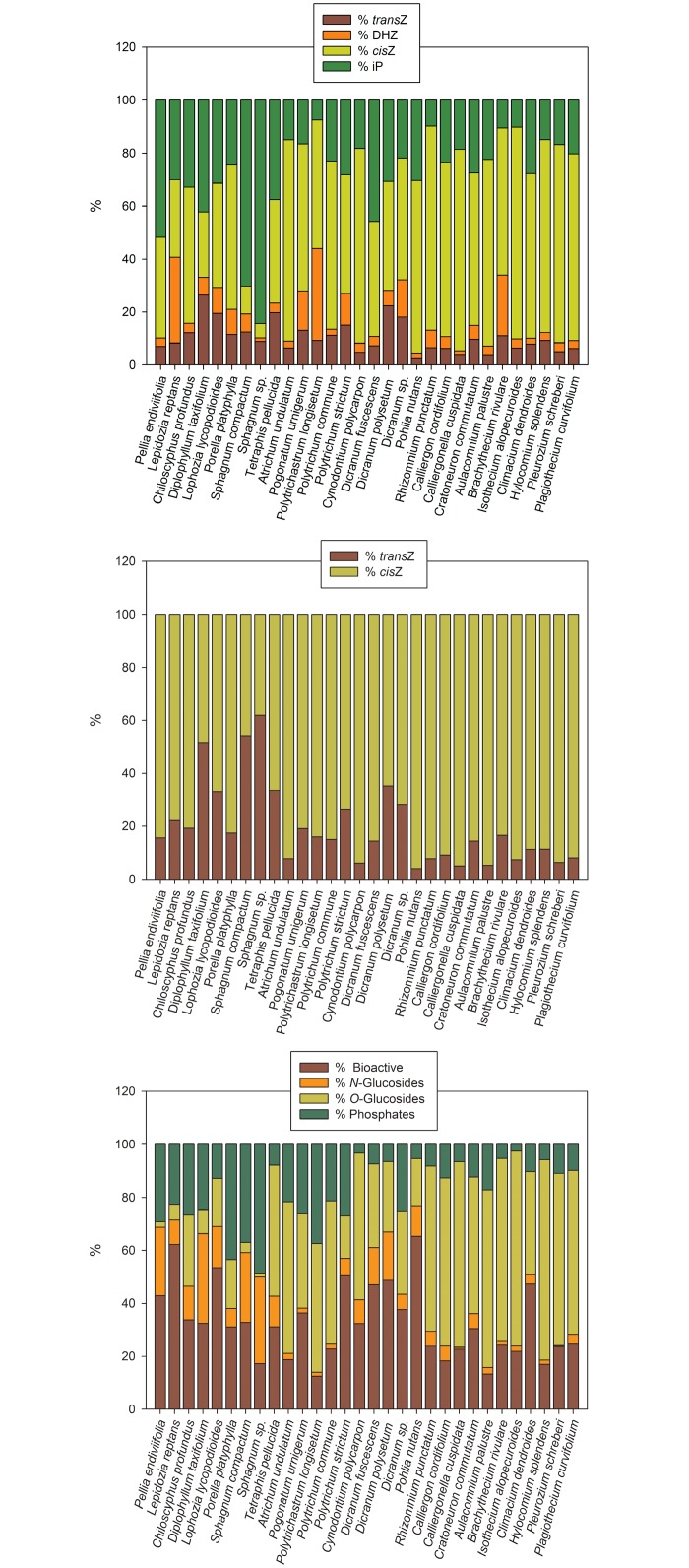
Proportions of particular cytokinin (CK) groups to the whole CK contents in bryophytes. **A.** Proportions of *trans*-zeatin (*trans*Z), dihydrozeatin (DHZ), *cis*-zeatin (*cis*Z) and *N* ^6^-(Δ^2^-isopentenyl)adenine (iP) types expressed as a percentage of the total CK pool; **B.** Proportional distribution between *trans*Z- and *cis*Z-type CKs; **C.** Proportions of bioactive forms (free bases and ribosides), *N*-glucosides (deactivation forms), *O*-glucosides (storage forms) and CK phosphates expressed as a percentage of the total CK pool. For details see S1 and S3 Tables.

In general, the *cis*Z- and iP-type CKs predominated. The *trans*Z-types were present in moderate concentrations (from 0.72 pmol/g FW in *Sphagnum compactum* to 18.57 pmol/g FW in *Polytrichastrum longisetum*) and DHZ forms only at very low levels towards the limit of detection (Fig 2A). There were only few exceptions to this generalization with relatively abundant DHZ derivatives such as in *Lepidozia reptans* (DHZ, 3.28 pmol/g FW), *Polytrichastrum longisetum* (DHZRMP, 63.87 pmol/g FW) and *Brachythecium rivulare* (DHZROG, 16.60 pmol/g FW). In twenty-seven species the amounts of *cis*Z-type CKs exceeded those of *trans*Z-types, in twenty-two of them very markedly being more than 3-fold higher. Only three species (*Diplophyllum taxifolium*, *Sphagnum compactum* and *Sphagnum* sp.) contained slightly higher (1.6-fold at the most) levels of *trans*Z-types compared to *cis*-zeatins (Fig 2B).

The bioactive CKs (especially free bases) and the *O*-glucosides were the most prevalent (Table 2 and S3 Table) making up most of the total CK pool in twenty-five species, usually contributing over 75% of the CK complement (Fig 2C). The CK ribosyl phosphates were also relatively common (with concentrations ranging from 0.90 pmol/g FW in *Isothecium alopecuroides* to 75.21 pmol/g FW in *Polytrichastrum longisetum*) and these CK ribosyl phosphates predominated in representatives of *Porellaceae* and *Sphagnaceae* families. The CK *N*-glucosides were present mostly at very low levels not exceeding 10% of the total CK pool. The proportional representation of CK *N*-glucoconjugates in the CK complement was generally higher in liverworts and *Sphagnaceae* family than in other mosses (Fig 2C).

**Table 2:** Distribution and endogenous levels (pmol/g FW) of different cytokinin (CK) types in bryophytes presented on the basis of their physiological function and conjugation status.

Division	Order	Family	Species	*trans*Z	*trans*ZR	DHZ	DHZR	*cis*Z	*cis*ZR	iP	iPR	∑ bioactive CKs	*trans*Z7G	*trans*Z9G	DZH9G	iP7G	iP9G	∑ N-Glucosides	*trans*ZOG	*trans*ZROG	DHZROG	*cis*ZOG	*cis*ZROG	∑ O-Glucosides	*trans*ZRMP	DZRMP	*cis*ZRMP	iPRMP	∑ CK phosphates
MARCHANTIOPHYTA (Liverworts)	Pelliales	Pelliaceae	*Pellia endiviifolia*	0,41	0,16	0,22	0,00	0,96	0,90	0,51	1,62	**4,78**	0,07	0,02	0,01	1,09	1,70	**2,88**	0,00	0,01	0,02	0,02	0,18	**0,23**	0,16	0,13	2,28	0,69	**3,26**
	Jungermaniales	Lepidoziaceae	*Lepidozia reptans*	0,36	0,16	3,28	0,06	0,77	0,94	1,57	0,70	**7,84**	0,19	0,01	0,25	0,45	0,25	**1,15**	0,02	0,12	0,11	0,03	0,49	**0,76**	0,18	0,38	1,45	0,84	**2,84**
	Jungermanniales	Lophocoleaceae	*Chiloscyphus profundus*	1,37	0,15	0,36	0,02	1,77	0,32	2,39	0,39	**6,78**	0,04	0,20	0,02	2,22	0,06	**2,55**	0,15	0,45	0,01	0,15	4,65	**5,40**	0,14	0,33	3,61	1,27	**5,35**
	Jungermanniales	Scapaniaceae	*Diplophyllum taxifolium*	1,04	0,21	0,28	0,22	0,67	1,85	0,46	0,42	**5,15**	0,06	0,10	0,01	5,11	0,11	**5,39**	0,25	0,20	0,18	0,04	0,73	**1,39**	2,34	0,39	0,59	0,65	**3,97**
	Jungermanniales	Lophoziaceae	*Lophozia lycopodioides*	1,08	0,14	0,73	0,31	0,28	2,98	0,99	0,48	**7,00**	0,02	0,25	0,02	1,52	0,22	**2,03**	0,36	0,12	0,13	0,02	1,75	**2,38**	0,61	0,07	0,06	0,95	**1,68**
	Porellales	Porellaceae	*Porella platyphylla*	0,53	0,08	0,25	0,01	1,82	0,59	1,47	1,05	**5,81**	0,14	0,27	0,16	0,53	0,21	**1,31**	0,10	0,69	1,36	0,02	1,27	**3,45**	0,36	0,01	6,56	1,19	**8,12**
BRYOPHYTA (Mosses)	Sphagnales	Sphagnaceae	*Sphagnum compactum*	0,50	0,05	0,06	0,09	0,10	0,13	0,17	0,76	**1,88**	0,01	0,01	0,01	1,24	0,25	**1,51**	0,05	0,00	0,01	0,01	0,15	**0,22**	0,10	0,20	0,20	1,62	**2,12**
	Sphagnales	Sphagnaceae	*Sphagnum sp*.	0,41	0,19	0,04	0,01	0,11	0,27	0,37	0,43	**1,84**	0,05	0,08	0,05	3,24	0,06	**3,48**	0,09	0,01	0,03	0,01	0,01	**0,16**	0,12	0,01	0,14	4,93	**5,20**
	Tetraphidales	Tetraphidaceae	*Tetraphis pellucida*	0,80	0,18	0,81	0,05	2,46	1,63	9,20	3,40	**18,53**	0,07	0,18	0,06	6,45	0,13	**6,89**	1,70	8,62	1,16	0,31	17,67	**29,46**	0,16	0,13	1,15	3,21	**4,65**
	Polytrichales	Polytrichaceae	*Atrichum undulatum*	0,93	0,40	0,23	0,06	3,10	3,81	2,21	1,23	**11,97**	0,11	0,15	0,17	1,07	0,02	**1,52**	0,31	1,85	0,45	1,02	33,00	**36,61**	0,38	0,72	7,71	5,03	**13,84**
	Polytrichales	Polytrichaceae	*Pogonatum urnigerum*	0,87	0,25	0,15	0,06	1,87	4,40	4,97	2,95	**15,52**	0,06	0,22	0,08	0,56	0,26	**1,18**	0,89	5,18	1,21	0,03	29,56	**36,86**	0,23	0,08	7,58	6,64	**14,53**
	Polytrichales	Polytrichaceae	*Polytrichastrum longisetum*	2,08	0,18	1,01	0,21	9,19	2,99	5,78	1,80	**23,24**	0,03	0,09	0,21	1,98	0,18	**2,48**	0,55	0,76	0,69	0,07	4,36	**6,44**	2,98	3,10	3,21	2,74	**12,03**
	Polytrichales	Polytrichaceae	*Polytrichum commune*	2,21	0,19	0,88	0,25	8,30	3,97	6,47	2,87	**25,14**	0,03	0,18	0,07	2,57	0,12	**2,98**	0,98	11,36	4,57	0,97	79,69	**97,57**	3,61	63,87	4,68	3,05	**75,21**
	Polytrichales	Polytrichaceae	*Polytrichum strictum*	2,85	0,51	1,37	0,47	5,90	4,38	1,95	4,87	**22,29**	0,04	0,16	0,13	0,71	0,14	**1,18**	0,82	1,59	0,33	0,07	19,03	**21,83**	2,13	6,90	4,51	2,55	**16,10**
	Dicranales	Dicranaceae	*Cynodontium polycarpon*	0,99	0,25	0,38	0,06	11,63	0,49	3,67	0,49	**17,97**	0,06	0,14	0,19	4,37	0,23	**4,99**	0,83	0,23	0,82	0,33	28,49	**30,71**	0,18	0,16	0,08	1,43	**1,85**
	Dicranales	Dicranaceae	*Dicranum fuscescens*	1,43	0,09	0,49	0,09	3,61	0,41	6,06	1,81	**14,00**	0,01	0,07	0,03	3,92	0,15	**4,17**	0,10	0,34	0,17	0,01	8,77	**9,40**	0,15	0,10	0,17	1,79	**2,21**
	Dicranales	Dicranaceae	*Dicranum polysetum*	0,99	0,15	0,38	0,02	2,43	0,20	2,64	1,14	**7,95**	0,05	1,83	0,44	0,25	0,42	**2,98**	0,29	0,31	0,03	0,35	3,37	**4,35**	0,03	0,08	0,37	0,59	**1,06**
	Dicranales	Dicranaceae	*Dicranum sp*.	2,08	0,14	0,33	0,14	2,70	0,41	1,52	0,98	**8,29**	0,02	0,05	0,07	0,56	0,57	**1,27**	0,26	0,56	0,18	0,39	5,49	**6,88**	0,88	2,37	1,13	1,21	**5,59**
	Bryales	Mniaceae	*Pohlia nutans*	1,03	0,16	0,46	0,13	32,53	1,78	5,99	3,61	**45,70**	0,05	0,05	0,01	2,27	5,71	**8,09**	0,22	0,24	0,58	0,03	11,35	**12,41**	0,17	0,08	0,18	3,36	**3,80**
	Bryales	Mniaceae	*Rhizomnium punctatum*	0,82	0,19	0,76	0,03	6,52	4,06	1,10	0,97	**14,45**	0,24	0,20	2,03	0,88	0,10	**3,45**	1,04	1,41	1,17	3,07	31,25	**37,94**	0,06	0,05	1,93	2,93	**4,96**
	Hypnales	Amblystegiaceae	*Calliergon cordifolium*	1,95	0,40	0,93	0,89	5,74	1,56	3,09	3,27	**17,84**	0,14	0,77	0,21	1,96	3,54	**6,62**	0,29	3,48	3,20	2,48	67,08	**76,52**	0,57	0,13	2,84	16,56	**20,10**
	Hypnales	Amblystegiaceae	*Calliergonella cuspidata*	0,86	0,16	0,08	0,03	4,41	0,33	6,74	2,02	**14,62**	0,02	0,07	0,03	0,25	0,09	**0,46**	0,15	1,24	0,60	2,02	41,20	**45,22**	0,09	0,13	1,09	2,93	**4,24**
	Hypnales	Amblystegiaceae	*Cratoneuron commutatum*	0,26	0,09	0,19	0,04	0,53	0,73	0,36	1,17	**3,38**	0,04	0,05	0,01	0,52	0,02	**0,63**	0,05	0,49	0,23	0,01	4,94	**5,72**	0,13	0,13	0,29	0,82	**1,37**
	Bryales	Aulacomniaceae	*Aulacomnium palustre*	0,84	0,17	0,78	0,02	10,66	1,16	1,96	3,45	**19,03**	0,04	0,12	0,13	2,57	0,63	**3,49**	0,62	3,73	3,59	4,19	83,81	**95,94**	0,09	0,13	0,80	23,49	**24,49**
	Hypnales	Brachytheciaceae	*Brachythecium rivulare*	1,01	0,41	0,30	0,03	10,91	0,77	3,33	1,27	**18,02**	0,08	0,11	0,06	0,73	0,04	**1,02**	3,37	1,93	16,60	1,25	28,41	**51,56**	1,34	0,10	0,06	2,47	**3,97**
	Hypnales	Brachytheciaceae	*Isothecium alopecuroides*	0,81	0,11	0,22	0,02	4,10	0,18	2,13	0,26	**7,83**	0,05	0,01	0,08	0,40	0,15	**0,69**	0,01	1,21	0,87	0,54	23,70	**26,33**	0,08	0,05	0,05	0,72	**0,90**
	Hypnales	Climaciaceae	*Climacium dendroides*	1,26	0,30	0,36	0,00	9,30	0,72	5,28	1,91	**19,13**	0,05	0,09	0,04	1,15	0,06	**1,38**	0,23	0,82	0,32	1,00	13,44	**15,81**	0,43	0,18	0,69	2,84	**4,14**
	Hypnales	Hylocomiceae	*Hylocomium splendens*	0,47	0,12	0,21	0,03	2,42	0,22	4,12	1,10	**8,71**	0,04	0,12	0,01	0,58	0,07	**0,82**	0,14	3,62	1,27	0,49	33,33	**38,84**	0,22	0,06	0,90	1,79	**2,97**
	Hypnales	Hylocomiaceae	*Pleurozium schreberi*	0,57	0,04	0,54	0,06	4,02	0,27	4,16	1,12	**10,77**	0,01	0,03	0,03	0,09	0,05	**0,21**	0,05	1,35	0,78	0,41	27,08	**29,67**	0,25	0,14	2,34	2,25	**4,98**
	Hypnales	Plagiotheciaceae	*Plagiothecium curvifolium*	0,57	0,17	0,98	0,05	5,23	0,64	5,53	1,24	**14,41**	0,08	0,41	0,11	1,19	0,42	**2,21**	0,30	1,62	0,46	1,32	32,62	**36,32**	0,46	0,18	1,57	3,53	**5,74**

CKs are divided into four groups including bioactive forms (free bases and ribosides), deactivation forms (*N*-glucosides), storage forms (*O*-glucosides) and CK phosphates. The results are shown as mean values; for complete data containing standard deviations of the means and coefficients of variance see S3 Table.

### Endogenous auxins

**Fig 3:**
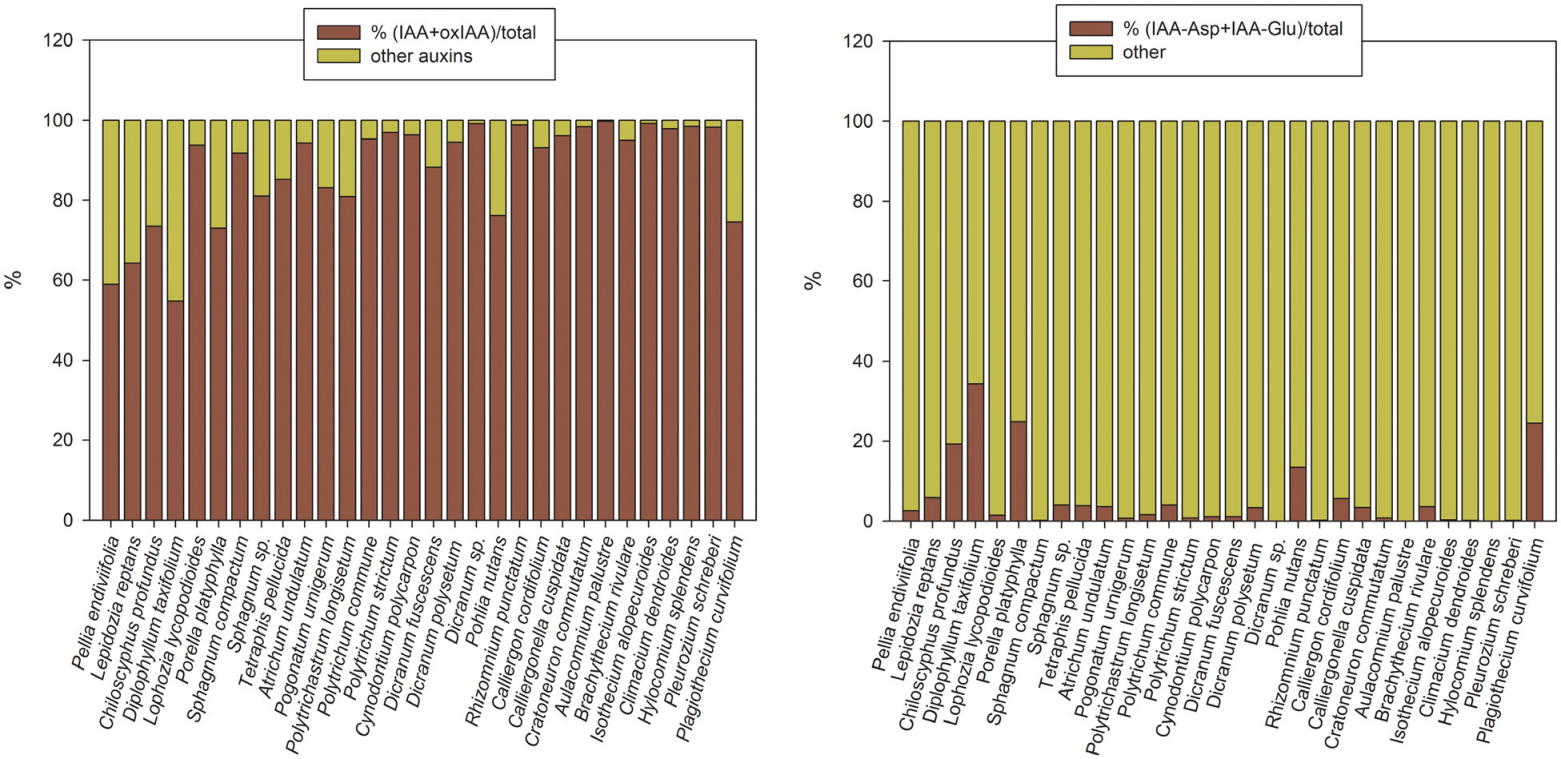
Proportions of particular auxin derivatives to the whole auxin contents in bryophytes. **A.** Proportions of indole-3-acetic acid (IAA) and 2-oxindole-3-acetic acid (oxIAA) expressed as a percentage of the total auxin pool; **B.** Proportions of IAA amino acid conjugates, IAA-aspartate (IAA-Asp) and IAA-glutamate (IAA-Glu), expressed as a percentage of the total auxin pool. For details see S4 Table.

The main auxins in all thirty analysed bryophyte species were free IAA ranging from 4.80 pmol/g FW (*Sphagnum* sp.) to 102.29 pmol/g FW (*Rhizomnium punctatum*) and its major primary catabolite 2-oxindole-3-acetic acid (oxIAA), occurring in similar concentrations (e.g. *Diplophyllum taxifolium*, *Sphagnum compactum*, *Cynodontium polycarpon*, *Dicranum fuscescens*) or frequently in higher amounts than IAA (e.g. 48-fold higher in *Polytrichum commune* or 41-fold higher in *Aulacomnium palustre*) (Table 3 and S4 Table). Indeed, these two indole derivatives made up a majority of the total auxin pool in all species, representing more than 80% of the auxin complement in twenty-three of them (Fig 3A). Interestingly, the proportion of oxIAA in the total auxin pool was generally lower in liverworts than in mosses. The glucosylesters of IAA (IAA-GE) and oxIAA (oxIAA-GE) were also present, however, with few exceptions (*Lepidozia reptans*, *Polytrichastrum longisetum*, *Pogonatum urnigerum*, *Tetraphis pellucida*) they did not contribute considerably to the auxin sum.

**Table 3:** Distribution and endogenous levels (pmol/g FW) of different auxin derivatives in bryophytes.

Division	Order	Family	Species	IAA	OxIAA	IAA-GE	OxIAA-GE	IAA-Asp	IAA-Glu	∑ IAA-Asp + IAA-Glu	IAN	IAM
MARCHANTIOPHYTA (Liverworts)	Pelliales	Pelliaceae	*Pellia endiviifolia*	77,46	128,46	0,32	9,25	4,73	4,35	**9,08**	0,00	124,85
	Jungermaniales	Lepidoziaceae	*Lepidozia reptans*	22,21	59,44	35,12	1,57	7,45	0,00	**7,45**	1,40	0,00
	Jungermanniales	Lophocoleaceae	*Chiloscyphus profundus*	29,18	41,87	0,83	4,51	3,36	15,26	**18,62**	0,00	1,75
	Jungermanniales	Scapaniaceae	*Diplophyllum taxifolium*	20,65	21,01	0,00	2,80	13,95	12,10	**26,05**	5,37	0,00
	Jungermanniales	Lophoziaceae	*Lophozia lycopodioides*	18,92	29,81	0,00	1,32	0,56	0,23	**0,79**	1,08	0,00
	Porellales	Porellaceae	*Porella platyphylla*	69,24	167,11	0,93	0,65	40,45	39,93	**80,38**	0,00	5,59
BRYOPHYTA (Mosses)	Sphagnales	Sphagnaceae	*Sphagnum compactum*	5,37	5,25	0,00	0,47	0,02	0,00	**0,02**	0,45	0,00
	Sphagnales	Sphagnaceae	*Sphagnum sp*.	4,80	6,58	0,66	0,24	0,10	0,48	**0,58**	0,00	1,19
	Tetraphidales	Tetraphidaceae	*Tetraphis pellucida*	17,00	41,49	0,76	5,98	2,68	0,00	**2,68**	0,71	0,00
	Polytrichales	Polytrichaceae	*Atrichum undulatum*	26,29	66,90	0,28	1,13	3,63	0,00	**3,63**	0,53	0,00
	Polytrichales	Polytrichaceae	*Pogonatum urnigerum*	16,35	785,67	2,44	1,80	6,32	27,69	**34,01**	0,00	1,22
	Polytrichales	Polytrichaceae	*Polytrichastrum longisetum*	11,63	67,71	0,00	1,44	0,27	0,39	**81,45**	0,42	0,00
	Polytrichales	Polytrichaceae	*Polytrichum commune*	9,05	98,70	0,00	23,06	1,49	0,67	**2,16**	0,26	0,00
	Polytrichales	Polytrichaceae	*Polytrichum strictum*	14,77	86,03	0,00	19,21	0,11	0,78	**0,89**	0,43	0,00
	Dicranales	Dicranaceae	*Cynodontium polycarpon*	41,11	39,58	0,00	1,12	0,62	0,29	**0,91**	1,02	0,00
	Dicranales	Dicranaceae	*Dicranum fuscescens*	15,95	17,76	0,00	1,49	0,19	0,24	**0,43**	2,58	0,00
	Dicranales	Dicranaceae	*Dicranum polysetum*	14,02	28,71	0,12	0,59	1,53	0,00	**1,53**	0,23	0,00
	Dicranales	Dicranaceae	*Dicranum sp*.	25,72	152,95	n. d.	0,45	0,09	0,12	**0,21**	0,83	0,00
	Bryales	Mniaceae	*Pohlia nutans*	29,96	34,91	0,86	3,86	4,95	6,46	**11,41**	0,00	4,21
	Bryales	Mniaceae	*Rhizomnium punctatum*	102,29	59,50	0,37	0,26	0,38	0,00	**0,38**	0,83	0,00
	Hypnales	Amblystegiaceae	*Calliergon cordifolium*	24,89	97,10	0,63	0,53	7,06	0,15	**130,36**	0,68	0,00
	Hypnales	Amblystegiaceae	*Calliergonella cuspidata*	15,14	89,96	0,09	0,34	3,77	0,00	**3,77**	0,06	0,00
	Hypnales	Amblystegiaceae	*Cratoneuron commutatum*	18,63	109,54	0,01	0,10	0,58	0,45	**1,03**	0,00	0,95
	Bryales	Aulacomniaceae	*Aulacomnium palustre*	44,35	1831,28	0,19	1,70	1,65	0,00	**1,65**	0,16	0,00
	Hypnales	Brachytheciaceae	*Brachythecium rivulare*	40,59	57,68	0,80	0,16	3,79	0,00	**3,79**	0,37	0,00
	Hypnales	Brachytheciaceae	*Isothecium alopecuroides*	23,75	77,12	0,12	0,28	0,25	0,00	**0,25**	0,16	0,00
	Hypnales	Climaciaceae	*Climacium dendroides*	18,59	37,77	0,20	0,61	0,11	0,00	**0,11**	0,31	0,00
	Hypnales	Hylocomiceae	*Hylocomium splendens*	10,37	95,84	0,31	0,83	0,10	0,00	**0,10**	0,37	0,00
	Hypnales	Hylocomiaceae	*Pleurozium schreberi*	15,30	112,92	0,44	0,76	0,21	0,00	**0,21**	0,84	0,00
	Hypnales	Plagiotheciaceae	*Plagiothecium curvifolium*	38,22	61,02	0,72	0,41	32,54	0,00	**32,54**	0,23	0,00

The results are shown as mean values; for complete data containing standard deviations of the means and coefficients of variance see S4 Table.

In vascular plants the IAA amino acid conjugates represent relatively abundant auxin forms [17,20,22], but were found to be much less common in bryophytes (Fig 3B). IAA-aspartate (IAA-Asp) and IAA-glutamate (IAA-Glu) were the sole IAA amino acid conjugates found in noticeable amounts (but lower than 5 pmol/g FW in twenty-three analyzed species and IAA-Glu being completely absent in fourteen species). Generally, the proportional representation of IAA-Asp and IAA-Glu in the auxin complement in liverworts exceeded that in mosses (Fig 3B, Table 3 and S4 Table).

Putative IAA precursors, indole-3-acetonitrile (IAN) and indole-3-acetamide (IAM), were detected. However, their occurrence was rather sporadic and, with rare exceptions (such as e.g. IAN in *Diplophyllum taxifolium* or IAM in *Pellia endiviifolia*, *Porella platyphylla* and *Pohlia nutans*), at very low amounts and close to the limit of detection (Table 3 and S4 Table).

### Other growth hormones (gibberellins, brassinosteroids)

Endogenous GAs (GA_4_, precursors GA_19_, GA_20_ and deactivation products GA_8_, GA_29_) as well as BRs were analyzed in all thirty bryophyte species. Although being detected in some of them, they occurred rather sporadically and mostly at low concentrations and close to the detection limit, which increased variability and reliability of the data. As no obvious trends in their quantities were found with respect to the phylogeny, the data are not included.

### Stress hormones

**Fig 4:**
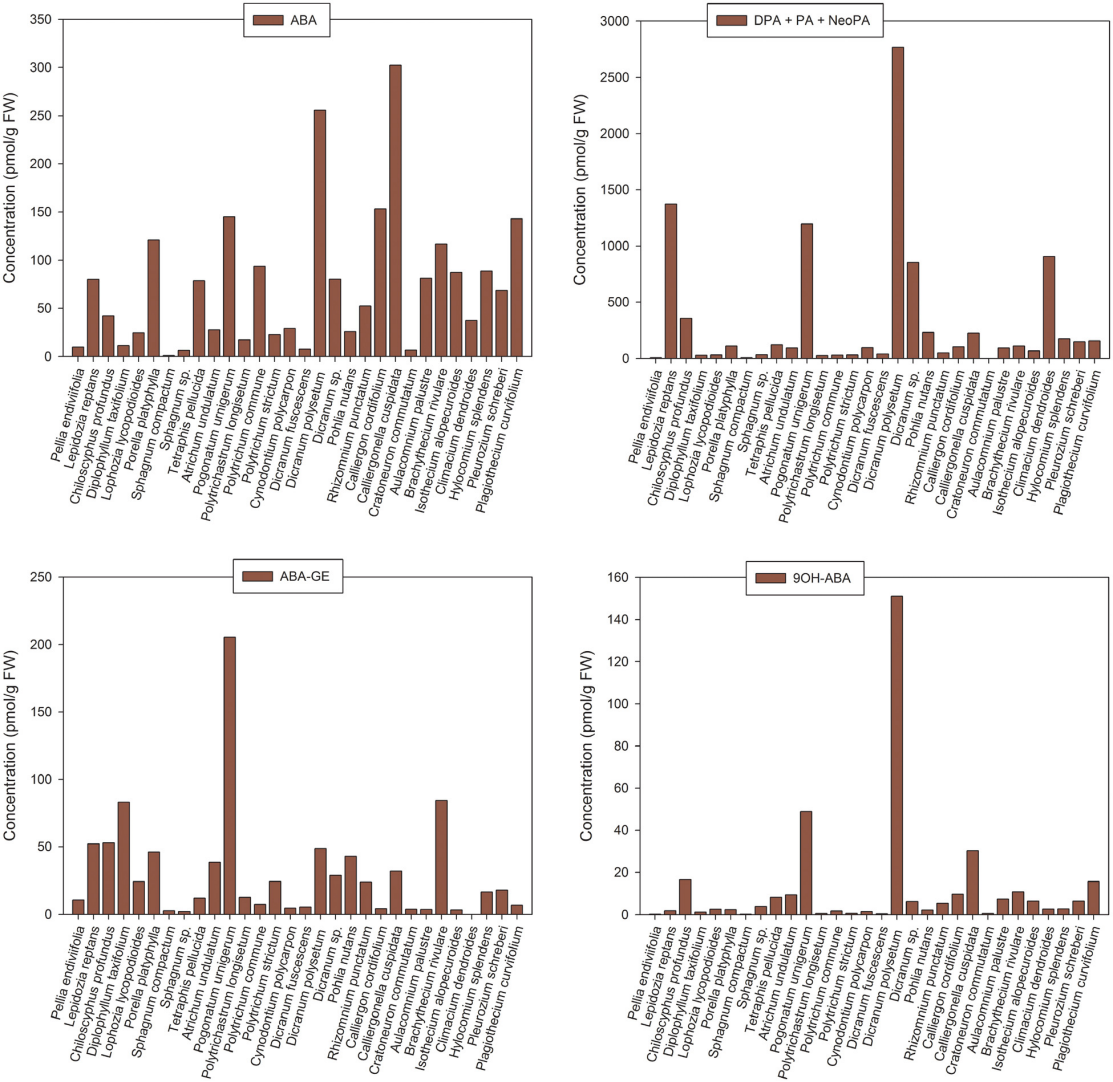
Endogenous concentration (pmol/g FW) of (A) abscisic acid and (B-D) its derivatives in bryophytes. **A.** Abscisic acid (ABA); **B.** Sum of ABA catabolites: dihydrophaseic acid (DPA) + phaseic acid (PA) + neophaseic acid (neoPA); **C.** ABA glucosylester (ABA-GE); **D.** 9-hydroxy-ABA (9OH-ABA). The results are shown as mean values; for complete data containing standard deviations of the means and coefficients of variance see S5 Table.

Abscisic acid was present in all thirty bryophyte samples with concentrations ranging from 1.02 pmol/g FW (*Sphagnum compactum*) to 302.22 pmol/g FW (*Calliergonella cuspidata*) (Fig 4A, Table 4 and S5 Table).

**Table 4:** Distribution and endogenous levels (pmol/g FW) of stress hormones abscisic acid (ABA), salicylic acid (SA) and jasmonic acid (JA) and their derivatives in bryophytes.

Division	Order	Family	Species	ABA	DPA	PA	ABA-GE	neoPA	9OH-ABA	SA	JA	JA-ILE
MARCHANTIOPHYTA (Liverworts)	Pelliales	Pelliaceae	*Pellia endiviifolia*	9,89	4,51	1,67	10,62	0,12	0,30	115,53	7,67	0,05
	Jungermaniales	Lepidoziaceae	*Lepidozia reptans*	79,93	1363,32	5,03	52,25	1,76	1,86	212,90	6,05	7,17
	Jungermanniales	Lophocoleaceae	*Chiloscyphus profundus*	41,98	321,94	12,88	52,97	21,10	16,61	410,63	3,85	3,70
	Jungermanniales	Scapaniaceae	*Diplophyllum taxifolium*	11,33	22,99	4,91	83,04	1,90	1,30	112,92	4,89	2,44
	Jungermanniales	Lophoziaceae	*Lophozia lycopodioides*	24,49	27,40	2,86	24,36	1,62	2,58	209,77	4,41	2,33
	Porellales	Porellaceae	*Porella platyphylla*	121,01	99,20	9,65	46,11	0,61	2,43	394,79	10,20	2,29
BRYOPHYTA (Mosses)	Sphagnales	Sphagnaceae	*Sphagnum compactum*	1,02	6,10	0,12	2,55	0,01	0,27	334,46	3,78	0,08
	Sphagnales	Sphagnaceae	*Sphagnum sp*.	6,39	32,13	0,50	2,04	1,37	3,91	65,28	1,20	0,08
	Tetraphidales	Tetraphidaceae	*Tetraphis pellucida*	78,61	70,45	48,43	11,99	1,94	8,18	154,97	29,75	4,29
	Polytrichales	Polytrichaceae	*Atrichum undulatum*	27,60	88,13	3,77	38,47	1,01	9,37	434,78	3,90	1,01
	Polytrichales	Polytrichaceae	*Pogonatum urnigerum*	93,67	29,40	0,79	7,32	0,54	1,85	90,85	5,55	0,09
	Polytrichales	Polytrichaceae	*Polytrichastrum longisetum*	22,76	28,95	2,17	24,44	0,90	0,70	205,39	7,54	0,31
	Polytrichales	Polytrichaceae	*Polytrichum commune*	17,31	25,01	1,18	12,54	0,08	0,62	135,88	7,54	0,43
	Polytrichales	Polytrichaceae	*Polytrichum strictum*	145,11	1124,01	71,47	205,60	1,25	48,89	134,86	78,80	1,14
	Dicranales	Dicranaceae	*Cynodontium polycarpon*	29,18	81,61	8,32	4,41	6,58	1,47	169,77	32,20	0,55
	Dicranales	Dicranaceae	*Dicranum fuscescens*	7,79	37,76	0,92	5,25	1,37	0,41	173,22	7,99	0,63
	Dicranales	Dicranaceae	*Dicranum polysetum*	255,72	2085,36	656,15	48,66	26,38	151,08	502,06	60,38	1,69
	Dicranales	Dicranaceae	*Dicranum sp*.	80,13	837,33	10,45	28,65	4,94	6,18	651,27	28,49	3,26
	Bryales	Mniaceae	*Pohlia nutans*	25,82	221,55	4,52	42,86	4,15	2,23	107,73	1,35	0,51
	Bryales	Mniaceae	*Rhizomnium punctatum*	52,31	40,74	8,39	24,02	2,07	5,36	300,09	28,89	0,96
	Hypnales	Amblystegiaceae	*Calliergon cordifolium*	153,22	59,53	26,28	4,10	16,83	9,70	502,12	12,77	2,28
	Hypnales	Amblystegiaceae	*Calliergonella cuspidata*	302,22	123,48	48,05	31,85	53,44	30,23	597,58	17,08	1,14
	Hypnales	Amblystegiaceae	*Cratoneuron commutatum*	6,67	0,96	1,18	3,65	0,46	0,62	127,12	4,41	0,11
	Bryales	Aulacomniaceae	*Aulacomnium palustre*	81,16	54,51	16,98	3,53	22,42	7,39	357,42	14,33	2,40
	Hypnales	Brachytheciaceae	*Brachythecium rivulare*	116,70	63,58	30,67	84,28	15,41	10,82	524,09	28,38	8,14
	Hypnales	Brachytheciaceae	*Isothecium alopecuroides*	87,23	50,24	11,27	3,11	5,54	6,39	304,31	12,25	1,86
	Hypnales	Climaciaceae	*Climacium dendroides*	37,15	888,97	10,98	0,33	6,23	2,68	210,60	17,09	1,90
	Hypnales	Hylocomiceae	*Hylocomium splendens*	88,67	144,88	12,78	16,63	15,51	2,77	224,48	15,64	0,52
	Hypnales	Hylocomiaceae	*Pleurozium schreberi*	68,68	105,79	13,35	17,93	29,21	6,37	453,01	4,76	0,19
	Hypnales	Plagiotheciaceae	*Plagiothecium curvifolium*	143,04	116,57	17,51	6,69	23,01	15,69	414,05	31,74	1,38

The results are shown as mean values; for complete data containing standard deviations of the means and coefficients of variance see S5 Table.

In addition, various ABA metabolites were determined (Fig 4B–4D). The most abundant ABA derivative was its physiologically inactive catabolite dihydrophaseic acid (DPA) (Fig 4B), which occurred in amounts ranging from 0.96 pmol/g FW (*Cratoneuron commutatum*) to 2085.36 pmol/g FW (*Dicranum polysetum*) often exceeding the concentration of free ABA (e.g. up to 24-fold in *Climacium dendroides*). The levels of other ABA catabolites, namely the weakly bioactive PA and inactive neophaseic acid (neoPA), were considerably reduced compared to DPA, up to 271- fold (*Lepidozia reptans*) and 900- fold (*Pogonatum urnigerum*), respectively (Fig 4B). The content of ABA glucosylester (ABA-GE) was higher than that of PA and neoPA in most cases (in 21 and 22 species, respectively), however, it did not usually exceed the level of DPA and free ABA (in 26 and 22 species, respectively) (Fig 4C). The concentration of another physiologically inactive ABA catabolite, 9-hydroxy-ABA (9OH-ABA), was rather low in the analyzed bryophyte samples (with only few exceptions such as *Chiloscyphus profundus*, *Pogonatum urnigerum*, *Dicranum polysetum* and *Calliergonella cuspidata*) and in any of these species it did not reach the contents of ABA and DPA (Fig 4D). No obvious trends in quantities of ABA or its metabolites were evident with respect to phylogeny.

**Fig 5:**
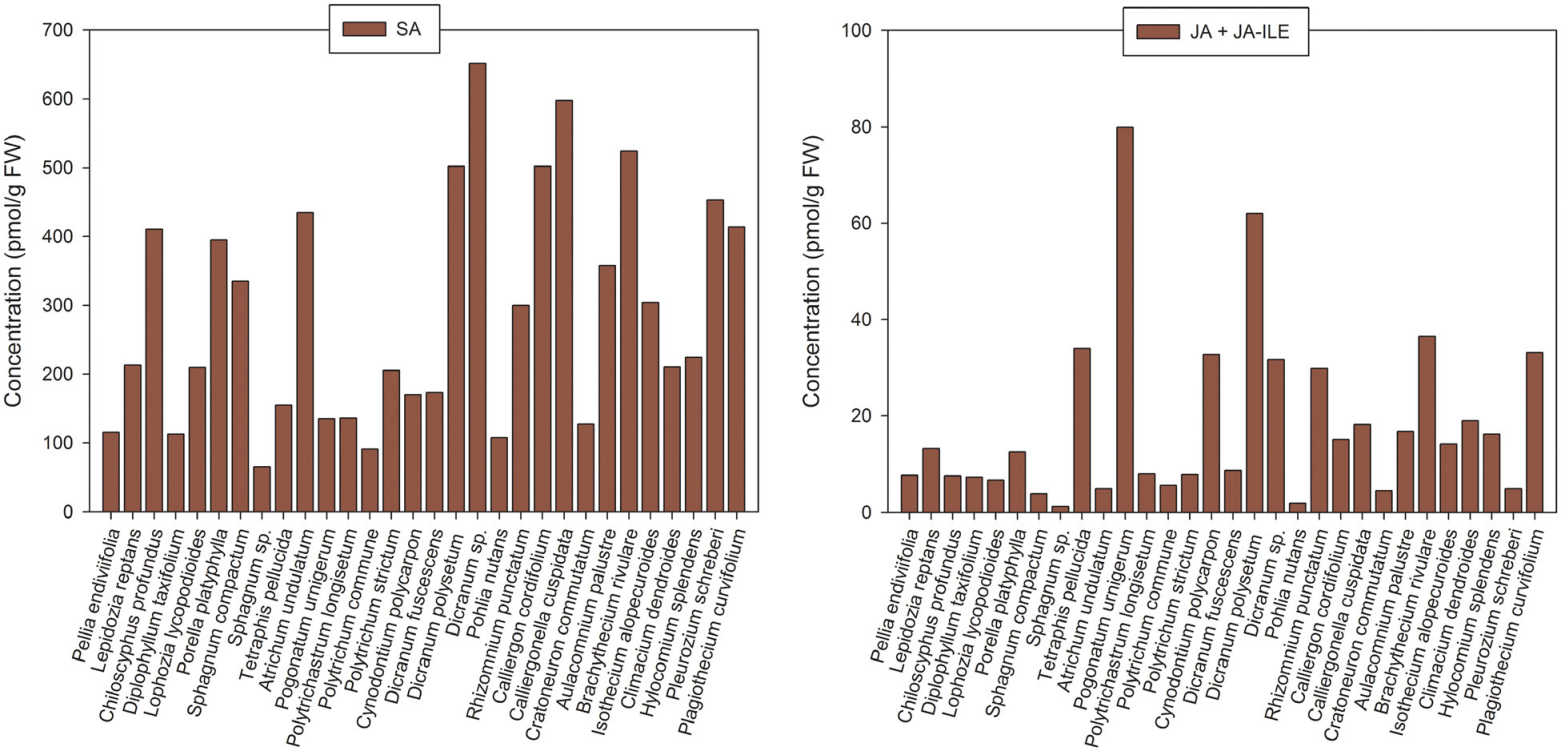
Endogenous concentration (pmol/g FW) of (A) salicylic acid (SA) and (B) jasmonic acid (JA) plus its isoleucine conjugate (JA-ILE) in bryophytes. **A.** Salicylic acid (SA); **B.** Sum of JA and JA-ILE. The results are shown as mean values; for complete data containing standard deviations of the means and coefficients of variance see S5 Table.

Salicylic acid (SA), a hormone involved in plant responses to biotic stresses, was relatively abundant in all screened bryophytes. Levels of SA ranged from tens of picomols (e.g. *Sphagnum sp*., 65.28 pmol/g FW) to hundreds of picomols (e.g. *Dicranum sp*., 651.27 pmol/g FW) showing no clear trends regarding the phylogeny of the species analyzed (Fig 5A, Table 4 and S5 Table).

Jasmonic acid (JA), a hormone regulating plant responses to both abiotic and biotic stresses, was detected in all bryophyte samples reaching concentrations from 1.20 pmol/g FW (*Sphagnum sp*.) to 78.80 pmol/g FW (*Pogonatum urnigerum*) (Fig 5B, Table 4 and S5 Table). The active JA metabolite, JA-isoleucine (JA-ILE), was less abundant than JA (except for *Lepidozia reptans*) and in a few cases (*Pellia endiviifolia*, *Sphagnum compactum*, *Sphagnum sp*., *Polytrichum commune*) it occurred only at very low amounts and close to the limit of detection. Again, no obvious trends with respect to phylogeny were noted (Fig 5B, Table 4 and S5 Table).

## Discussion

### Phytohormone profiling in bryophytes

The functioning of various classes of phytohormones in control of growth and development of seed plants is well known. Increasing evidence demonstrates an important role of plant hormones in coordination of growth and stress responses in other organisms including bryophytes [10,11,15]. However, there is little information regarding endogenous phytohormone profiling in bryophytes, with the available information being restricted mostly to the model mosses, *Physcomitrella patens* and, in lesser extent, *Funaria hygrometrica* ([10,15,23,24] and references therein).

The extensive screen presented here reveals a wide array of phytohormones found in bryophytes. Taking advantage of a high performance liquid chromatography electrospray tandem-mass spectrometry (HPLC-ESI-MS/MS) methodology, we detected over 40 different metabolic forms of phytohormones including growth hormones (CKs, auxins, GAs, BRs), stress hormones (ABA, JA, SA) and their conjugates, the most comprehensive survey of the bryophyte hormonome so far.

Our analyses revealed 26 native CKs in bryophytes, far more than previously described for mosses [25]. The predominance of *cis*Z- and iP-type CKs over *trans*Z- and DHZ-types corresponds well to the reported profiles of CKs in *Physcomitrella patens* [26–28]. Considerably more *cis*- than *trans*-zeatins were found in liverworts and mosses consistent with an earlier report [29] and the zeatins predominated in all but the Sphagnums. In contrast, iP-types were the major CK forms in tissue-cultrured *Funaria hygrometrica* [30] and *Physcomitrella patens* [31].

In contrast to vascular plants showing largely a strong glucoconjugation of CKs [13,22,32,33], very low levels of CK *N*-glucoconjugates were detected in almost all of the bryophytes. In general, the proportion of CK *N*-glucosides was higher in liverworts and the *Sphagnaceae* than in other mosses, in which they were either not detected at all (as for *Physcomitrella patens*; [26]) or found in very low concentrations not exceeding 10% of the total CK pool. It is possible that the absent or sparse *N*-glucosyltransferase pathway deactivating CKs in seed plants is substituted in bryophytes by enhanced formation of weakly active *cis*Z derivatives and/or by degradation by CK oxidase/dehydrogenase (CKX) as suggested based on detection of this enzyme activity in *Funaria hygrometrica* [34] and *Physcomitrella patens* tissues [26] as well as on revealing CKX EST’s in *Physcomitrella patens* [35].

It is well documented that the genomes of the „basal”land plants, such as *Physcomitrella patens*, contain members of gene families associated with biosynthesis, metabolism, transport and signaling of auxins [18,36], i.e. the complete auxin machinery seems to be present already in bryophytes. In all thirty bryophyte species the major auxins were free IAA and its principal oxidative catabolite, oxIAA. Free IAA was unequivocally identified in *Physcomitrella patens* as early as 30 years ago [37] and then shown to occur in liverworts, hornworts and mosses [38,39]. In vascular plants oxIAA has been shown to be an integral constituent of the auxin hormonome [40,41], the present report is the first record of oxIAA in bryophytes. Interestingly, proportional representation of oxIAA was lower in liverworts than in mosses suggesting less turnover of IAA in the former.

Conjugation of IAA to amino acids represents an important pathway for auxin homeostasis in higher plants [17,22,42]. In our analyses of bryophytes, IAA amino acid conjugates were not abundant and only IAA-Asp and IAA-Glu were found in some species. As for CK-glucoconjugates, the IAA amino acid conjugate complement in liverworts mostly exceeded that in mosses. The results suggest that liverworts prefer conjugation while mosses favour degradation strategies to maintain homeostasis. Our findings do not correspond to the data of Sztein et al. ([38,43]; reviewed in [17,44]) indicating that mosses employ a conjugation-hydrolysis strategy to control their auxin concentration, and liverworts mostly degradation. Evidence suggests that all bryophyte genomes express both metabolic and catabolic enzymes, but that prevailing environmental conditions determine opposite strategies for hormone turnover. It is noted that the bryophytes in our study were collected directly from natural conditions whereas samples from previous reports were from tissue culture. Interestingly, the auxin profile in our moss samples resembles that in seaweeds and algae, where also only few conjugates were detected ([14,45,46] and references therein).

Endogenous GAs were detected in some bryophytes but rather sporadically, mostly at low concentrations and without any obvious tendencies with respect to phylogeny. Our data correspond with their occurrence and/or functioning in liverworts, hornworts and mosses [11,15]. The role of GAs remains unknown in bryophytes and it is possible that the molecular structures of putative GA-like compounds in bryophytes differ strongly from those in seed plants so that neither their production nor specific effects have been discovered [10].

ABA together with its physiologically inactive catabolite, DPA, was relatively abundant in all thirty bryophyte species. Numerous other ABA catabolites (PA, neoPA and 9OH-ABA) as well as its storage glucosyl ester (ABA-GE) were detected in most of the samples. However, no trends with respect to phylogeny were evident. The production as well as physiological role of ABA in modulating cellular responses of bryophytes to environmental signals has been well documented ([9,11,15,24] and references therein). The occurrence of DPA, PA, neoPA, 9OH-ABA as well as ABA-GE reported here indicates that ABA is intensively metabolically degraded and/or conjugated in liverworts and mosses and that these processes may occur *via* biochemical pathways similar to those known for vascular plants.

Hormones involved in defense and stress responses (SA, JA and its active form, JA-ILE) were found in all species. Since literature data regarding the occurrence and physiological functions of these stress hormones in liverworts, hornworts and/or mosses is still rather scattered (for review see [11]), more findings are needed to understand their distribution and signal transduction mechanisms in the bryophytes. As for ABA, no obvious trends were observed with respect to the phylogeny.

### Comparison of the phytohormone profiles of liverworts and mosses

This study has allowed to contrast and compare the phytohormone profiles of liverworts and mosses which have featured rarely in hormone analyses in the plant kingdom. Recent transcriptome studies [47] place liverworts at the root of the land plant evolutionary tree of life. Our study shows clearly that, by the branch point, all the phytohormones familiar in the angiosperm lineage are already present through the bryophyta. Furthermore, very similar metabolic and catabolic spectra were recorded suggesting that the bryophytes represent a step in building-up a system of hormonal regulations in plants. Nevertheless, there are also some distinctions from the angiosperms. In particular, there is intensive production of *cis*Z-type CKs, whereas it is the *trans*Z family which predominates in the angiosperms. Auxin is removed from the system by oxidative degradation in all bryophytes, as appears to be the case for Arabidopsis [40]. Profiling indicates that conjugation of both CKs and auxins is present but weak in bryophytes, however, with some slight differences between liverworts and mosses. For example, the CK *N*-glucoconjugates are generally larger contributors to the total CK content in liverworts than in mosses. In the auxinome, the proportion of IAA amino acid conjugates, IAA-Asp and IAA-Glu, in the total auxin pool in liverworts generally exceeds that in mosses. Therefore, there are likely to be subtle differences in how homeostasis is managed between these two classes.

Among bryophytes, the liverworts are the earliest lineage sister to all other groups of land plants [47]. Only the liverworts, like vascular plants, synthetize and accumulate a myriad of isoprenoid compounds, which make them very unique. The isoprenoid (or terpenoid) pathway is one of the most important biosynthetic pathways in plants. Isoprenoids are the most numerous and structurally diverse group and represent the largest family of natural products [48,49]. Except for the isoprenoid biosynthetic pathways, also some physical and kinetic characteristics at the enzyme level in liverworts are similar to those in vascular plants [50–52]. Moreover, the liverworts have special oil bodies, where isoprenoid biosynthetic enzymes certainly operate [53]. The liverwort oil bodies are intracytoplasmatic secretory structures bound to a single membrane [54]. These structures have no subcellular equivalent in mosses and hornworts or in vascular plants. All these data for liverworts uniqueness converging to vascular plants might help explaining our findings that proportional representation of CK *N*-glucoconjugates in the CK complement as well as the proportion of IAA amino acid conjugates, IAA-Asp and IAA-Glu, in the total auxin pool in liverworts generally exceeds that in mosses. On the other hand, the observation of miRNA in *Pellia endiviifolia* shows a link between algae and liverworts because the same miRNAs exist in both but are not present in land plants [55].

## Conclusions

To summarize, the determined endogenous phytohormone contents suggest that evolution of bryophytes is associated with evolution of the hormonome. The profiles of plant growth hormones indicate that weak conjugation of both CKs and auxins, intensive production of *cis*Z-type CKs as well as strong oxidative degradation of auxins, seem to be common traits in all bryophytes. The apparent differences in conjugation and/or degradation strategies of growth hormones between liverworts and mosses might potentially show a hidden link between vascular plants and liverworts. On the other hand, the complement of stress hormones in bryophytes probably correlate rather with a strategy of life and prevailing environmental conditions at the point of sample collection than with evolutionary aspects. Evidently, this comprehensive survey indicates the validity of experimentation done on bryophytes for phytohormone evolution and extends our knowledge of these ubiquitous and fascinating organisms.

## Supporting Information

S1 Table List of analyzed bryophyte species with localities and dates of collection. Systematic arrangement was done according to Encyclopedia of Life (available from http://www.eol.org, accessed 15 January 2014).
(DOCX)

S2 Table List of abbreviations. Abbreviations for cytokinin derivatives adopted and modified according to [21].
(DOC)

S3 Table Complete list of distribution and endogenous levels (pmol/g FW) of different cytokinin (CK) types in bryophytes. Mean values of two independent measurements are shown including standard deviation (SD) of the means and coefficient of variance (CV).
(XLSX)

S4 Table Complete list of distribution and endogenous levels (pmol/g FW) of different auxin derivatives in bryophytes. Mean values of two independent measurements are shown including standard deviation (SD) of the means and coefficient of variance (CV).
(XLSX)

S5 Table Complete list of distribution and endogenous levels (pmol/g FW) of stress hormones abscisic acid (ABA), salicylic acid (SA) and jasmonic acid (JA) and their derivatives in bryophytes. Mean values of two independent measurements are shown including standard deviation (SD) of the means and coefficient of variance (CV).
(XLSX)
