# Supplementary material for: Phytohormone Profiling across the Bryophytes
Source: PLoS One. 2015 May 14;10(5):e0125411. doi: 10.1371/journal.pone.0125411 (PMC4431756; doi:10.1371/journal.pone.0125411)
Supplement: S1 Table — Systematic arrangement was done according to Encyclopedia of Life (available from http://www.eol.org, accessed 15 January 2014). (DOCX) [file pone.0125411.s001.docx]

**Table S1. List of analysed bryophyte species with localities and dates of collection. Systematic arrangement was done according to Encyclopedia of Life (available from** [**http://www.eol.org**](http://www.eol.org)**, accessed 15 January 2014).**

| **Division** | **Order, Family** | **Species** | **Locality** | **Date** |
| --- | --- | --- | --- | --- |
| **MARCHANTIOPHYTA (Liverworts)** | Pelliales, Pelliaceae | *Pellia endiviifolia* (Dicks.) Dumort. | Central Bohemia, Křivoklátsko, “U Eremita”, wet rocks, ca 250 m alt., 50°1'1.684"N, 13°51'40.623"E | 19. 4. 2011 |
|  | Jungermaniales, Lepidoziaceae | *Lepidozia reptans* (L.) Dumort. | Central Bohemia, Křivoklátsko, Klíčava springs, wet natural depression, ca 400 m alt., 50°8'40.683"N, 13°49'45.663"E | 19. 4. 2011 |
|  | Jungermaniales, Lophocoleaceae (Jungermanniaceae s. l.) | *Chiloscyphus profundus* (Nees) J.J. Engel & R.M. Schust. | Central Bohemia, Křivoklátsko, Řevničov, forest area, ca 450 m alt., 50°8'31.585"N, 13°49'16.865"E | 19. 4. 2011 |
|  | Jungermaniales, Scapaniaceae | *Diplophyllum taxifolium* (Wahlenb.) Dum. | Krkonoše Mts., Kozí hřbety, ca 1340 m alt., 50°43'59.442"N, 15°39'26.366"E | 8. 8. 2012 |
|  | Jungermaniales, Lophoziaceae (s. s.; Scapaniaceae s. l.) | *Lophozia lycopodioides* (Wallr.) Cogn. | Krkonoše Mts., Kozí hřbety, ca 1340 m alt., 50°43'59.442"N, 15°39'26.366"E | 8. 8. 2012 |
|  | Porellales, Porellaceae | *Porella platyphylla* (L.) Pfeiff. | Central Bohemia, Křivoklátsko, “U Eremita”, wet rocks, ca 250 m alt., 50°1'1.684"N, 13°51'40.623"E | 19. 4. 2011 |
| **BRYOPHYTA (Mosses)** | Sphagnales, Sphagnaceae | *Sphagnum compactum* Lam. et DC. | Krkonoše Mts., Kozí hřbety, ca 1340 m alt., 50°43'59.442"N, 15°39'26.366"E | 8. 8. 2012 |
|  | Sphagnales, Sphagnaceae | *Sphagnum* sp. | Central Bohemia, Křivoklátsko, Klíčava springs, wet natural depression, ca 400 m alt., 50°8'40.683"N, 13°49'45.663"E | 19. 4. 2011 |
|  | Tetraphidales, Tetraphidaceae | *Tetraphis pellucida* Hedw. | Central Bohemia, Křivoklátsko, Klíčava springs, wet natural depression, ca 400 m alt., 50°8'43.289"N, 13°49'40.763"E | 19. 4. 2011 |
|  | Polytrichales, Polytrichaceae | *Atrichum undulatum* (Hedw.) P. Beauv. | Central Bohemia, Křivoklátsko, Řevničov, forest area, ca 450 m alt., 50°8'31.585"N, 13°49'16.865"E | 19. 4. 2011 |
|  | Polytrichales, Polytrichaceae | *Pogonatum urnigerum* (Hedw.) P. Beauv. | Krkonoše Mts., Kozí hřbety, ca 1340 m alt., 50°43'59.442"N, 15°39'26.366"E | 8. 8. 2012 |
|  | Polytrichales, Polytrichaceae | *Polytrichastrum longisetum* (Bridel) G. L. Smith | Krkonoše Mts., Kozí hřbety, ca 1340 m alt., 50°43'59.442"N, 15°39'26.366"E | 8. 8. 2012 |
|  | Polytrichales, Polytrichaceae | *Polytrichum commune* Hedw. | Central Bohemia, Křivoklátsko, Řevničov, forest area, ca 450 m alt., 50°8'31.585"N, 13°49'16.865"E | 19. 4. 2011 |
|  | Polytrichales, Polytrichaceae | *Polytrichum strictum* (Menzies ex) Brid. | Krkonoše Mts., Kozí hřbety, ca 1340 m alt., 50°43'59.442"N, 15°39'26.366"E | 8. 8. 2012 |
|  | Dicranales, Dicranaceae | *Cynodontium polycarpon* (Hedw.) Schimp. | Krkonoše Mts., Kozí hřbety, ca 1340 m alt., 50°43'59.442"N, 15°39'26.366"E | 8. 8. 2012 |
|  | Dicranales, Dicranaceae | *Dicranum fuscescens* Sm. | Krkonoše Mts., Kozí hřbety, ca 1340 m alt., 50°43'59.442"N, 15°39'26.366"E | 8. 8. 2012 |
|  | Dicranales, Dicranaceae | *Dicranum polysetum* Sw. | Central Bohemia, Křivoklátsko, Řevničov, forest area, ca 450 m alt., 50°8'31.585"N, 13°49'16.865"E | 19. 4. 2011 |
|  | Dicranales, Dicranaceae | *Dicranum* sp. | Central Bohemia, Křivoklátsko, Svatá village, Vraní skála, ca 530 m alt., 49°55'40.067"N, 13°56'28.919"E | 28.5.2012 |
|  | Bryales, Mniaceae | *Pohlia nutans* (Hedw.) Lindb. | Central Bohemia, Křivoklátsko, Řevničov, forest area, ca 450 m alt., 50°8'31.585"N, 13°49'16.865"E | 19. 4. 2011 |
|  | Bryales, Mniaceae | *Rhizomnium punctatum* (Hedw.) T. Kop. | Central Bohemia, Křivoklátsko, “U Eremita”, wet rocks, ca 250 m alt., 50°1'1.684"N, 13°51'40.623"E | 19. 4. 2011 |
|  | Hypnales, Amblystegiaceae | *Calliergon cordifolium* (Hedwig) Kindb. | Central Bohemia, Křivoklátsko, Klíčava springs, wet, natural depression, ca 400 m alt., 50°8'40.683"N, 13°49'45.663"E | 19. 4. 2011 |
|  | Hypnales, Amblystegiaceae | *Calliergonella cuspidata* (Hedw.) Loeske | Central Bohemia, Křivoklátsko, Klíčava springs, wet natural depression, ca 400 m alt., 50°8'43.289"N, 13°49'40.763"E | 19. 4. 2011 |
|  | Hypnales, Amblystegiaceae | *Cratoneuron commutatum* (Brid.) G. Roth. | Central Bohemia, Křivoklátsko, “U Eremita”, wet rocks, ca 250 m alt., 50°1'1.684"N, 13°51'40.623"E | 19. 4. 2011 |
|  | Bryales, Aulacomniaceae | *Aulacomnium palustre* (Hedw.) Schwägr. | Central Bohemia, Křivoklátsko, Klíčava springs, wet natural depression, ca 400 m alt., 50°8'43.289"N, 13°49'40.763"E | 19. 4. 2011 |
|  | Hypnales, Brachytheciaceae | *Brachythecium rivulare* Schimp. | Central Bohemia, Křivoklátsko, “U Eremita”, wet rocks, ca 250 m alt., 50°1'1.684"N, 13°51'40.623"E | 19. 4. 2011 |
|  | Hypnales, Brachytheciaceae | *Isothecium alopecuroides* (Dubois) Isov. | Central Bohemia, Křivoklátsko, “U Eremita”, wet rocks, ca 250 m alt., 50°1'1.684"N, 13°51'40.623"E | 19. 4. 2011 |
|  | Hypnales, Climaciaceae | *Climacium dendroides* (Hedw.) F.Weber & D.Mohr. | Central Bohemia, Křivoklátsko, Klíčava springs, wet natural depression, ca 400 m alt., 50°8'40.683"N, 13°49'45.663"E | 19. 4. 2011 |
|  | Hypnales, Hylocomiceae | *Hylocomium splendens* Hedw. | Central Bohemia, Křivoklátsko, Řevničov, forest area, ca 450 m alt., 50°8'31.585"N, 13°49'16.865"E | 19. 4. 2011 |
|  | Hypnales, Hylocomiaceae | *Pleurozium schreberi* (Brid.) Mitt. | Central Bohemia, Křivoklátsko, Řevničov, forest area, ca 450 m alt., 50°8'31.585"N, 13°49'16.865"E | 19. 4. 2011 |
|  | Hypnales, Plagiotheciaceae | *Plagiothecium curvifolium* Schlieph. ex Limpr. | Central Bohemia, Křivoklátsko, Řevničov, forest area, ca 450 m alt., 50°8'31.585"N, 13°49'16.865"E | 19. 4. 2011 |
