# Supplementary material for: Phytohormone Profiling across the Bryophytes
Source: PLoS One. 2015 May 14;10(5):e0125411. doi: 10.1371/journal.pone.0125411 (PMC4431756; doi:10.1371/journal.pone.0125411)
Supplement: S2 Table — Abbreviations for cytokinin derivatives adopted and modified according to [21]. (DOC) [file pone.0125411.s002.doc]

**Table S2. List of abbreviations for cytokinin derivatives**

9OH-ABA, 9-hydroxy-abscisic acid;

ABA, abscisic acid;

ABA-GE, abscisic acid glucosyl ester;

BR, brassinosteroid;

*cis*Z, *cis-*zeatin;

*cis*ZR, *cis-*zeatin riboside;

*cis*Z7G, *cis*-zeatin 7-glucoside;

*cis*Z9G, *cis*-zeatin 9-glucoside;

*cis*ZOG, *cis*-zeatin *O*-glucoside;

*cis*ZROG, *cis*-zeatin 9-riboside *O*-glucoside;

*cis*ZRMP, *cis*-zeatin 9-riboside-5΄-monophosphate;

CK, cytokinin;

CKX, cytokinin oxidase/dehydrogenase;

DHZ, dihydrozeatin;

DHZR, dihydrozeatin 9-riboside;

DHZ7G, dihydrozeatin 7-glucoside;

DHZ9G, dihydrozeatin 9-glucoside;

DHZROG, dihydrozeatin 9-riboside *O*-glucoside;

DHZRMP, dihydrozeatin 9-riboside-5΄-monophosphate;

DPA, dihydrophaseic acid;

GA, gibberellin;

IAA, indole-3-acetic acid;

IAA-Asp, indole-3-acetic acid aspartate;

IAA-GE, indole-3-acetic acid glucosyl ester;

IAA-Glu, indole-3-acetic acid glutamate;

IAM, indole-3-acetamide;

IAN, indole-3-acetonitrile;

iP, *N*^6^-(∆^2^-isopentenyl)adenine;

iPR, *N*^6^-(∆^2^-isopentenyl)adenine 9-riboside;

iP7G, *N*^6^-(∆^2^-isopentenyl)adenine 7-glucoside;

iP9G, *N*^6^-(∆^2^-isopentenyl)adenine 9-glucoside;

iPRMP, *N*^6^-(∆^2^-isopentenyl)adenine 9-riboside-5΄-monophosphate;

oxIAA, 2-oxindole-3-acetic acid;

oxIAA-GE, 2-oxindole-3-acetic acid glucosyl ester;

JA, jasmonic acid;

JA-ILE, jasmonic acid isoleucine;

neoPA, neophaseic acid;

PA, phaseic acid;

SA, salicylic acid;

*trans*Z, *trans*-zeatin;

*trans*ZR, *trans*-zeatin 9-riboside;

*trans*Z7G, *trans*-zeatin 7-glucoside;

*trans*Z9G, *trans*-zeatin 9-glucoside;

*trans*ZOG, *trans*-zeatin *O*-glucoside;

*trans*ZROG, *trans*-zeatin 9-riboside *O*-glucoside;

*trans*ZRMP, *trans*-zeatin 9-riboside-5΄-monophosphate;

(abbreviations for cytokinins adopted and modified according to Kamínek et al. 2000).
